# Comparison of Aging Performances and Mechanisms: Super-Durable Fire-Resistant “Xuan Paper” Versus Chinese Traditional Xuan Paper

**DOI:** 10.3390/molecules30020263

**Published:** 2025-01-10

**Authors:** Li-Ying Dong, Ying-Jie Zhu, Jin Wu, Han-Ping Yu

**Affiliations:** 1 State Key Laboratory of High Performance Ceramics and Superfine Microstructure, Shanghai Institute of Ceramics, Chinese Academy of Sciences, Shanghai 200050, China; lydong@mail.sic.ac.cn (L.-Y.D.); wujin@mail.sic.ac.cn (J.W.); yuhanping@mail.sic.ac.cn (H.-P.Y.); 2 Center of Materials Science and Optoelectronics Engineering, University of Chinese Academy of Sciences, Beijing 100049, China

**Keywords:** hydroxyapatite, nanowire, fire-resistant, Xuan paper, durability, accelerated aging

## Abstract

Paper is a thin nonwoven material made from cellulose fibers as the main raw material together with some additives. Paper is highly flammable, leading to the destruction of countless precious ancient books, documents, and art works in fire disasters. In recent years, researchers have made a lot of efforts in order to obtain more durable and fire-retardant paper. Owing to the successful synthesis of ultralong hydroxyapatite (HAP) nanowires as a new kind of inorganic nanofiber material, it becomes possible to develop a new kind of super-durable and fire-resistant paper. Recently, the authors’ research group prepared a new kind of fire-resistant “Xuan paper” consisting of ultralong HAP nanowires. In this article, the super-durable fire-resistant “Xuan paper” based on ultralong HAP nanowires and the traditional Xuan paper based on cellulose fibers were evaluated by the accelerated aging method for 1200 days at 105 °C in air, which is the equivalent of 10,000 years of natural aging in the ambient environment. The aging mechanism of the traditional Xuan paper was further investigated by studying the fiber length/width and their distributions, morphology, infrared spectroscopy, thermogravimetric analysis, H–nuclear magnetic resonance spectra, and C–nuclear magnetic resonance spectra of cellulose fibers before and after the accelerated aging. The durability, properties, and mechanism of the fire-resistant “Xuan paper” based on ultralong HAP nanowires during the accelerated aging were studied. The experiments reveal the reasons for the deteriorated properties and reduced durability by aging of the traditional Xuan paper based on cellulose fibers, and the mechanism for the super-durability and excellent performances of the fire-resistant “Xuan paper” based on ultralong HAP nanowires during the accelerated aging process.

## 1. Introduction

Paper is a thin nonwoven material made from cellulose fibers as the main raw material together with some additives such as fillers, sizing agents, retention aids, defoamers, and bleaching agents. Paper is one of the greatest inventions in the world, and it has greatly promoted the rapid development of human civilization. The history of paper can be traced back to ancient times. The earliest paper appeared more than two thousand years ago during the Western Han Dynasty in China [1]. In today’s society, paper is widely used in various fields, in addition to being applied for writing and printing. However, there are some problems facing the cellulose-fiber-based traditional paper. For example, papermaking consumes a large amount of wood and causes environmental pollution. In addition, paper made from the mechanical pulping process contains lignin which can form a yellow material in the presence of light and oxygen, and that is why the traditional paper yellows with age. Traditional paper is also at risk of acid decay because cellulose itself produces acids. Furthermore, a key problem for the cellulose-based traditional paper is its high flammability and easy destruction in fire.

Among various kinds of paper, the traditional Chinese Xuan paper is an excellent representative of traditional handmade paper [2,3]. The traditional Chinese Xuan paper originated in Jing Country, Anhui Province in eastern China. The invention of the traditional Xuan paper can be traced back to the Tang Dynasty, China about 1500 years ago [4]. In 2009, the traditional handicraft of making Xuan paper was inscribed on the Representative List of the Intangible Cultural Heritage of Humanity by the Educational, Scientific, and Cultural Organization of the United Nations [5]. The excellent durability of the traditional Chinese Xuan paper is attributed to its unique raw materials and handmade manufacturing process which involves more than 100 steps [6]. The main raw material of the traditional Chinese Xuan paper is elm bark fibers of *Pteroceltis tatarinowii* with good flexibility and a high even degree, and limestone particles are deposited on the surface of elm bark fibers, which can neutralize acids produced by the hydrolysis of plant fibers and from the environment [6]. In addition, the complex handmade production process under mild treatment conditions results in the least chemical damage to plant fibers. The traditional Chinese Xuan paper exhibits superior properties such as durability, ink wetting, wet deformation, and resistance to insects and mildew, which make it the most durable paper in the world with a recognized name of “the king of paper that lasts for 1000 years” [4]. The traditional Xuan paper is mainly used for painting arts and calligraphy works. The good durability of the traditional Xuan paper contributes to the long-term preservation of precious paintings and calligraphy works. Although the traditional Xuan paper is the most durable paper in the world, there are some problems facing the traditional Xuan paper with an organic origin during the long preservation process, for example, yellowing and decreased mechanical properties. In addition, a key problem for the traditional Xuan paper is its high flammability, which is a threat to the safe preservation of paintings and calligraphy works based on the traditional Xuan paper [4]. During humanity’s long history, numerous precious calligraphy and painting works and books were ruined in fire disasters.

To solve the problems facing the traditional paper, it is important to explore new materials as the building materials for the production of more durable and fire-retardant paper [1]. In recent years, efforts of researchers have been devoted to developing more durable fire-resistant paper. For example, synthetic inorganic fibers were used as raw materials of the fire-resistant paper. Hydroxyapatite (HAP) is the main inorganic component of human bones and teeth and has a high biocompatibility [7,8]. In recent years, many HAP nanostructured materials with different morphologies were synthesized using various strategies, for instance, nanoparticles [9,10], nanorods [11,12,13,14], nanowires [15,16,17,18], nanotubes [19,20,21,22], nanosheets [23,24,25,26], and mesoporous microspheres [27,28,29,30], which were investigated for applications in various fields. Among various morphologies of HAP nanostructured materials, ultralong HAP nanowires have unique properties and are promising for a variety of applications. Ultralong HAP nanowires have good biocompatibility, environmental friendliness, high flexibility, high whiteness, and excellent resistance to fire and high temperature. Ultralong HAP nanowires have promising applications for the construction of various flexible fire-resistant materials [1].

As a result of considerable effort in recent years, the author’s research group developed a novel synthetic method (the calcium oleate precursor solvothermal/hydrothermal method) [31,32]. By using the calcium oleate precursor solvothermal/hydrothermal method, we successfully synthesized highly flexible ultralong hydroxyapatite (HAP) nanowires with diameters of about 10 nm, lengths of several hundred micrometers, and aspect ratios of more than 10,000 [1,32]. Among various inorganic fibers, ultralong HAP nanowires are the only inorganic nanofibers containing a large number of hydroxyl functional groups. Hydroxyl functional groups are a key factor for forming the hydrogen bonding between fibers and for gaining the high mechanical properties of paper. In addition, ultralong HAP nanowires have ultrahigh aspect ratios and small diameters, leading to high flexibility. The high whiteness of ultralong HAP nanowires without bleaching is another important advantage for the construction of the fire-resistant paper. This new nanofiber material can be used for the development of a new kind of fire-resistant paper to tackle the problems facing the traditional cellulose-fiber-based flammable paper. Recently, many kinds of functional fire-resistant paper based on ultralong HAP nanowires were prepared and investigated for applications in various fields [1,33]. Among various kinds of functional fire-resistant paper based on ultralong HAP nanowires, a new kind of fire-resistant “Xuan paper” was developed using ultralong HAP nanowires by the authors’ research group, which has unique ink wetting properties, superior anti-mildew performance, super-durability, and excellent resistance to both high temperature and fire [4].

The main differences between the traditional Xuan paper and the fire-resistant “Xuan paper” synthesized by the authors are summarized below: (1) The raw materials used for the production of the two kinds of paper are different. The traditional Xuan paper uses elm bark fibers of *Pteroceltis tatarinowii* as the main raw material, but the fire-resistant “Xuan paper” synthesized by the authors adopts ultralong HAP nanowires as the raw material. (2) The manufacturing processes of the two types of paper are different. The traditional Chinese Xuan paper is produced by the handmade manufacturing process which involves more than 100 steps, which takes a long period of time (about 1~2 years). However, the fire-resistant “Xuan paper” synthesized by the authors is produced by only a few steps and takes a short period of time (only 2~3 days). (3) The properties of the two types of paper are different. Although the traditional Xuan paper is the most durable paper in the world, there are some problems facing the traditional Xuan paper with the organic origin during the long preservation process, for example, yellowing and decreased mechanical properties. In addition, a key problem for the traditional Xuan paper is its high flammability, which is a threat to the safe preservation of paintings and calligraphy works based on the traditional Xuan paper. In contrast, the fire-resistant “Xuan paper” synthesized by the authors has unique excellent resistance to both high temperature and fire. (4) The service life times of the two types of paper are different. The traditional Xuan paper has a service life of about 1000 years; however, the fire-resistant “Xuan paper” synthesized by the authors has a much longer service life of up to more than 10,000 years.

In this work, the effects of aging time up to 10,000 years of natural aging in the ambient environment on the durability and properties of the traditional Chinese Xuan paper based on cellulose fibers, and the fire-resistant “Xuan paper” based on ultralong HAP nanowires were investigated for the first time. The traditional Chinese Xuan paper based on cellulose fibers and the fire-resistant “Xuan paper” based on ultralong HAP nanowires were evaluated by the accelerated aging method in the lab for 1200 days, which is equivalent to 10,000 years of natural aging in the ambient environment. The aging mechanism of the traditional Xuan paper was further investigated by studying the fiber length/width and their distributions, morphology, infrared spectroscopy, thermogravimetric analysis, H–nuclear magnetic resonance spectra, and C–nuclear magnetic resonance spectra of cellulose fibers before and after the accelerated aging. Furthermore, the durability, properties, and mechanism of the fire-resistant “Xuan paper” based on ultralong HAP nanowires during the accelerated aging process were studied. The experiments reveal the reasons for the aging of the traditional Xuan paper based on cellulose fibers and for the super-durability and excellent performances of the fire-resistant “Xuan paper” based on ultralong HAP nanowires during the accelerated aging process.

## 2. Results and Discussion

### 2.1. The Performance and Durability of the Fire-Resistant “Xuan Paper” During the Accelerated Heat Aging Process

In this work, the accelerated heat aging method was adopted to evaluate the performance and durability of paper according to the standard of the Technical Association of the Pulp and Paper Industry of USA (TAPPI) [34]. In the accelerated heat aging experiment, the paper was placed in an electric oven maintained at a constant temperature of 105 °C for a certain time. Accelerated heating aging for 72 h corresponds to natural aging for 25 years in the ambient environment. After continuous accelerated heat aging for different times, the properties of paper sheets were measured.

We first discuss the performance and durability of the fire-resistant “Xuan paper” based on ultralong HAP nanowires during the accelerated heat aging process. The fire-resistant “Xuan paper” is prepared using ultralong HAP nanowires, glass fibers, and an inorganic adhesive developed in our laboratory. The base weight and thickness of the as-prepared fire-resistant “Xuan paper” based on ultralong HAP nanowires are 71 g m^−2^ and 91 μm, respectively. The tightness and bulk of the fire-resistant “Xuan paper” based on ultralong HAP nanowires are 0.78 g cm^−3^ and 1.28 cm^3^ g^−1^, respectively. The whiteness and the tensile strength of the as-prepared fire-resistant “Xuan paper” based on ultralong HAP nanowires are as high as 92% and 8.3 N/15 mm without the accelerated heat aging.

**Figure 1:**
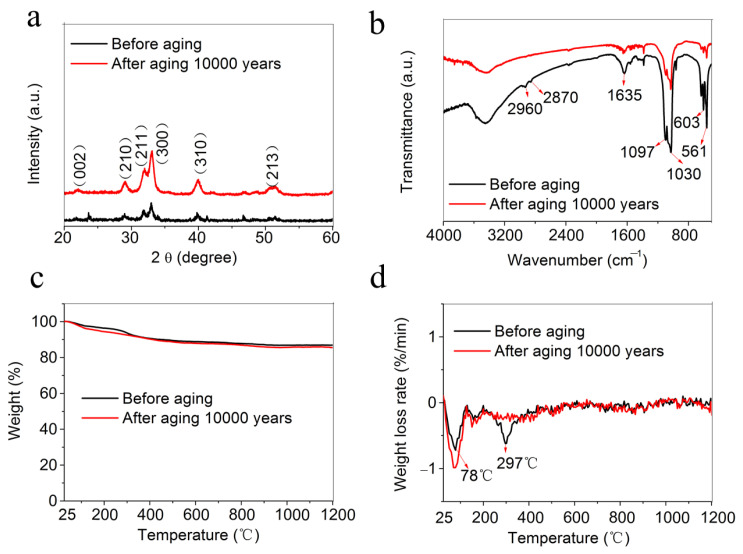
Characterization of the fire-resistant “Xuan paper” based on ultralong HAP nanowires before and after the continuous accelerated heat aging for 1200 days, which is equivalent to 10,000 years of natural aging in the ambient environment: (**a**) XRD patterns; (**b**) Fourier-transform infrared (FTIR) spectra; (**c**) thermogravimetric (TG) curves; and (**d**) the derivate thermogravimetric (DTG) curves.

**Figure 2:**
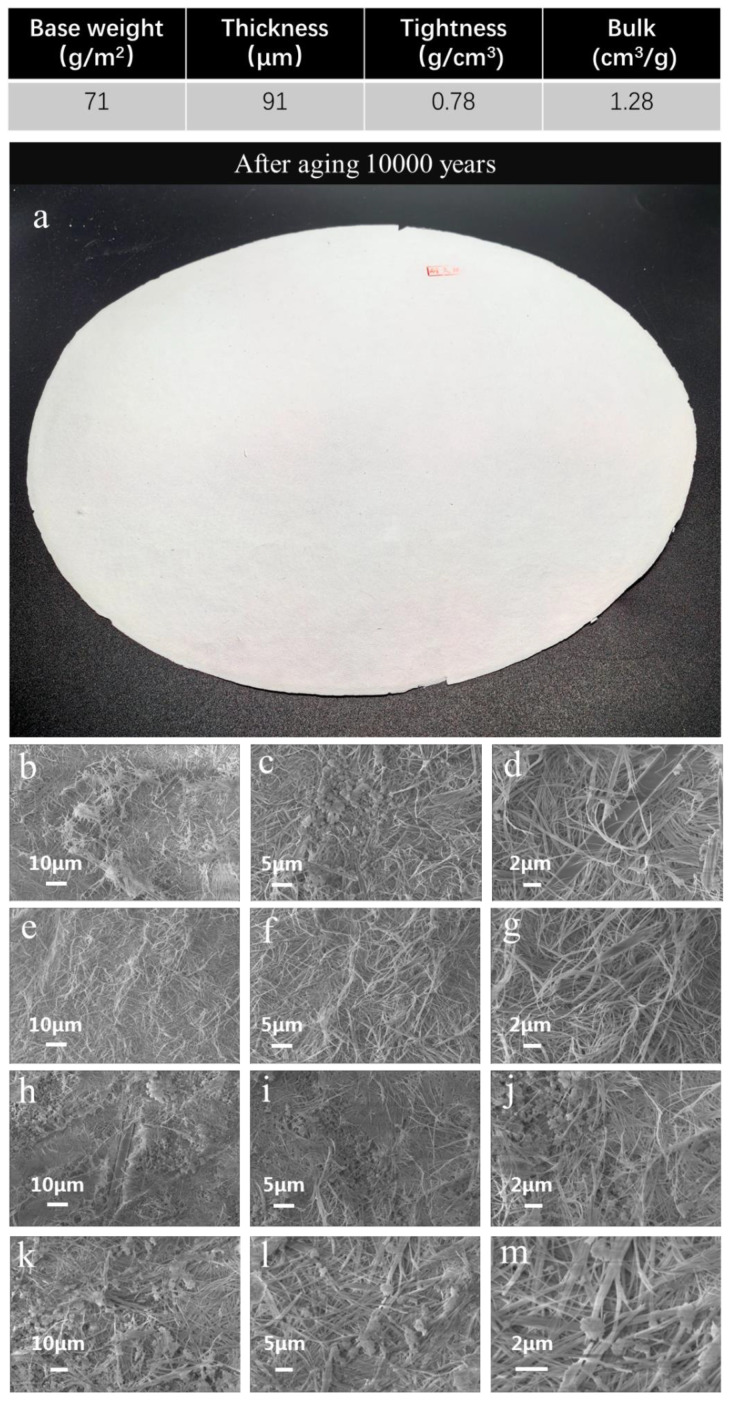
Characterization of the fire-resistant “Xuan paper” based on ultralong HAP nanowires after the continuous accelerated heat aging for 1200 days: (**a**) a digital image showing the appearance of the fire-resistant “Xuan paper” after the continuous accelerated heat aging for 1200 days; (**b**–**g**) SEM images of the top surface (**b**–**d**) and the bottom surface (**e**–**g**) of the fire-resistant “Xuan paper” without the accelerated heat aging; and (**h**–**m**) SEM images of the top surface (**h**–**j**) and the bottom surface (**k**–**m**) of the fire-resistant “Xuan paper” after the continuous accelerated heat aging for 1200 days.

Compared with the traditional Xuan paper based on cellulose fibers, the fire-resistant “Xuan paper” based on ultralong HAP nanowires exhibits superior properties and durability during the simulated heat aging process. After the simulated heat aging equivalent to 10,000 years of natural aging in the ambient environment, no obvious changes are observed in the composition, structure, and properties of the fire-resistant “Xuan paper” such as the crystal phase, morphology of ultralong HAP nanowires, whiteness, and tensile strength, as shown in Figure 1 and Figure 2. Ultralong HAP nanowires as the main building material of the fire-resistant “Xuan paper” can be well-preserved in terms of structure, composition, wire length, whiteness, and mechanical properties after the accelerated heat aging for 1200 days which is equivalent to 10,000 years of natural aging in the ambient environment. These experimental results reveal the reason for the high performance and super-durability of the fire-resistant “Xuan paper” during the accelerated heat aging process.

Figure 1a shows the X-ray diffraction (XRD) patterns of the fire-resistant “Xuan paper” before and after the accelerated heat aging for 1200 days, which is equivalent to 10,000 years of natural aging in the ambient environment. The two XRD patterns have similar X-ray diffraction peaks, which can be indexed to the crystal phase of hydroxyapatite (JCPDF no. 09-0432), indicating that the main building material is ultralong HAP nanowires. Figure 1b,c shows the Fourier-transform infrared (FTIR) spectra and thermogravimetric (TG) curves of the fire-resistant “Xuan paper” before and after the accelerated heat aging equivalent to 10,000 years of natural aging. Both the FTIR spectra of the fire-resistant “Xuan paper” before and after the accelerated heat aging for 1200 days show the absorption peaks at 1030 cm^−1^ and 1097 cm^−1^, corresponding to the stretching mode of the PO_4_^3−^ group, and the absorption peaks located at 603 cm^−1^ and 561 cm^−1^ are attributed to the bending mode of the O-P-O of the PO_4_^3−^ group, suggesting that the structure of ultralong HAP nanowires within the fire-resistant “Xuan paper” does not change after the accelerated heat aging for 1200 days. But the absorption peak at around 1635 cm^−1^, which belongs to the residual oleate groups on the surface of ultralong HAP nanowires, is weakened after the aging process. This may be ascribed to the degradation of oleate groups during the aging process. It is consistent with the thermal analysis results. Although both the TG curves of the fire-resistant “Xuan paper” before and after the accelerated heat aging for 1200 days exhibit similar and small weight loss, their derivative thermogravimetric (DTG) curves are different (Figure 1d). One can see that the DTG curve of the fire-resistant “Xuan paper” before the accelerated heat aging shows two obvious peaks at 78 °C and 297 °C, which are ascribed to the loss of absorbed water and the oxidation of oleate groups, respectively. However, only the peak at about 78 °C can be observed in the DTG curve of the fire-resistant “Xuan paper” after the accelerated heat aging for 1200 days, and the peak belonging to the oxidation of oleate groups disappears. It can be concluded that the accelerated heat aging for 1200 days of the fire-resistant “Xuan paper” does not change the structure of ultralong HAP nanowires but promote the degradation of residual oleate groups.

In addition, the appearance of the fire-resistant “Xuan paper” after the accelerated heat aging for 1200 days, equivalent to 10,000 years of natural aging, in the ambient environment is intact and very white in color, as shown in Figure 2a. Figure 2b–d shows SEM images of the top surface of the fire-resistant “Xuan paper” based on ultralong HAP nanowires without the accelerated heat aging, and Figure 2e–g shows SEM images of the bottom surface of the fire-resistant “Xuan paper” without the accelerated heat aging. One can see a large number of ultralong HAP nanowires as the main building material on both the top surface and the bottom surface of the fire-resistant “Xuan paper” without the accelerated heat aging. Furthermore, there are some particles in some areas on the surface of the fire-resistant “Xuan paper”; these particles are the inorganic adhesive added in the paper during the preparation process to enhance the mechanical properties of the fire-resistant “Xuan paper”, as shown in Figure 2c. In addition, glass fibers with micrometer-sized diameters are observed in some locations, which can significantly reinforce the structure and enhance the mechanical properties of the fire-resistant “Xuan paper”, as shown by one thick and long glass fiber in Figure 2d. Figure 2h–j presents SEM images of the top surface of the fire-resistant “Xuan paper” after the accelerated heat aging equivalent to 10,000 years of natural aging, and Figure 2k–m shows SEM images of the bottom surface of the fire-resistant “Xuan paper” after the accelerated heat aging equivalent to 10,000 years of natural aging. Similar to the fire-resistant “Xuan paper” without the accelerated heat aging, no obvious change is observed for the fire-resistant “Xuan paper” after the accelerated heat aging equivalent to 10,000 years of natural aging, and ultralong HAP nanowires, glass fibers, and inorganic adhesive particles can be observed on both the top surface and the bottom surface of the fire-resistant “Xuan paper”. No obvious change is observed for ultralong HAP nanowires as the main building material in the fire-resistant “Xuan paper” even after the accelerated heat aging equivalent to as long as 10,000 years of natural aging. The experimental results indicate that ultralong HAP nanowires as the main building material of the fire-resistant “Xuan paper” have essentially no change before and after the accelerated heat aging equivalent to 10,000 years of natural aging, which can be explained by the inorganic nature and high stability of ultralong HAP nanowires, which is very different from organic materials. The experimental results reveal the intrinsic reasons and mechanism for the super-durability and excellent performance of the fire-resistant “Xuan paper” based on ultralong HAP nanowires during the accelerated aging process.

The whiteness, whiteness retention rate, tensile strength, and retention rate of tensile strength of the fire-resistant “Xuan paper” based on ultralong HAP nanowires before and after the accelerated heat aging equivalent to 10,000 years of natural aging in the ambient environment were investigated. The experimental results show that, after the accelerated heat aging equivalent to 10,000 years of natural aging, the whiteness of the fire-resistant “Xuan paper” slightly decreases from 92% to 90.7% with a retention rate of as high as 98.6%. Similarly, the tensile strength of the fire-resistant “Xuan paper” based on ultralong HAP nanowires is reduced from 8.3 to 7.9 N/15mm after the accelerated heat aging equivalent to 10,000 years of natural aging with a high retention rate of 95.2%. The experimental results indicate that the fire-resistant “Xuan paper” based on ultralong HAP nanowires exhibits excellent performance and still maintains superior properties even after the accelerated heat aging equivalent to 10,000 years of natural aging in the ambient environment.

To summarize the above, the fire-resistant “Xuan paper” exhibits excellent structural and physiochemical stability even after the accelerated heat aging equivalent to 10,000 years of natural aging. No significant difference between the fire-resistant “Xuan paper” before and after aging could be identified in both appearance and SEM observations. This is because it is composed of all inorganic components: ultralong HAP nanowires, glass fibers, and inorganic adhesive particles. Although a small amount of residual oleate groups is adsorbed on the surface of ultralong HAP nanowires during the nanowire synthesis, they will not affect the overall architecture of the fire-resistant “Xuan paper” and are removed during the long-term aging process. As a result, the fire-resistant “Xuan paper” maintains high retention rates in terms of both whiteness and tensile strength after the accelerated heat aging equivalent to 10,000 years of natural aging. These anti-aging advantages, combined with its unique advantage of non-flammability and high thermal stability, make the novel kind of fire-resistant “Xuan paper” promising for the application in the protection of valuable art works and important paper documents for long-term preservation and protection from fire damage.

Moreover, it should be noted that the cleaning extent of ultralong HAP nanowires has an obvious effect on the properties and durability of the fire-resistant “Xuan paper” during the accelerated heat aging process. In a previous work, ultralong HAP nanowires used for the preparation of the fire-resistant “Xuan paper” were washed with ethanol and deionized water three times, respectively, and ultralong HAP nanowires were partially clean, and there were still some oleate groups adsorbed on the surface of ultralong HAP nanowires [4]. In this work, much cleaner ultralong HAP nanowires processed by washing with ethanol and deionized water many times are adopted for the preparation of the fire-resistant “Xuan paper”. The experimental results indicate that the fire-resistant “Xuan paper” consisting of cleaner ultralong HAP nanowires exhibits better properties and more durable performance compared with those of the fire-resistant “Xuan paper” consisting of ultralong HAP nanowires with more oleate groups adsorbed on nanowire surfaces [4].

### 2.2. Aging Performance and Mechanism of the Traditional Chinese Xuan Paper

**Figure 3:**
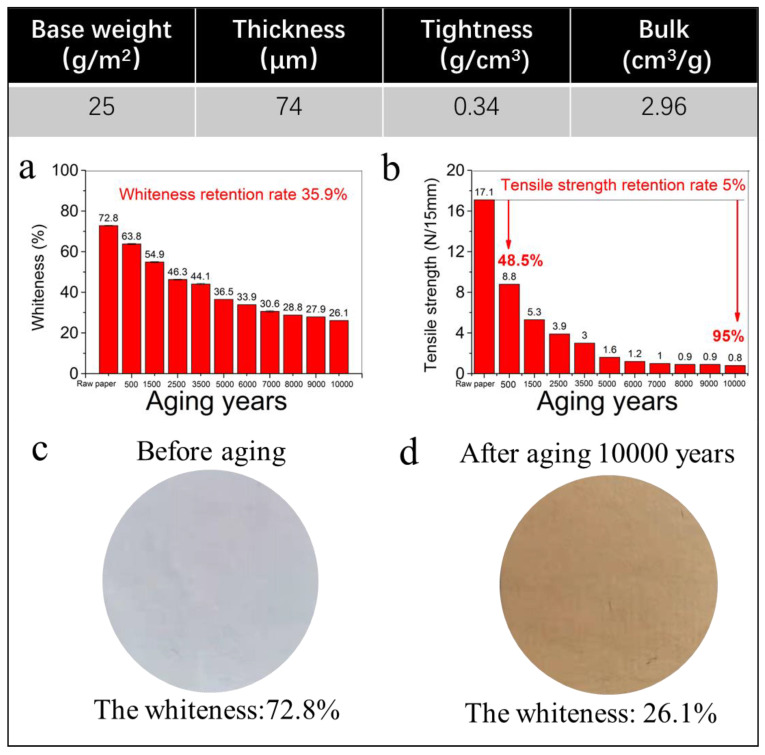
The performance evaluation of the traditional Chinese Xuan paper based on cellulose fibers before and after the continuous accelerated heat aging for up to 1200 days: (**a**) whiteness versus aging time of the traditional Xuan paper; (**b**) tensile strength versus aging time of the traditional Xuan paper; and (**c**,**d**) digital images of the traditional Xuan paper before (**c**) and after (**d**) the continuous accelerated heat aging for 1200 days.

It is well-known that the paper-based books, calligraphy, and painting works in museums and homes turn yellow and become brittle during the long-term preservation process, indicating the decreasing whiteness and mechanical properties of paper. The experimental results of the traditional Chinese Xuan paper based on cellulose fibers before and after different accelerated heat aging times of up to 1200 days, which is equivalent to 10,000 years of natural aging, are shown in Figure 3. Figure 3a shows the results of whiteness versus aging time of the traditional Xuan paper before and after different accelerated heat aging times of up to 1200 days, which is equivalent to 10,000 years of natural aging. The whiteness of the traditional Xuan paper is ~72.8% before the accelerated heat aging. With increasing the aging time, the whiteness of the traditional Xuan paper continues to decrease. When the continuous accelerated heat aging time is 600 days, which is equivalent to 5000 years of natural aging, the whiteness of the traditional Xuan paper decreases from 72.8% to 36.5% with a retention rate of 50.1%. When the continuous accelerated heat aging time is 1200 days, which is equivalent to 10,000 years of natural aging, the whiteness of the traditional Xuan paper further decreases to 26.1% with a low retention rate of only 35.9%, indicating that the traditional Xuan paper exhibits a severe yellowing phenomenon during the continuous accelerated heat aging process, as shown in Figure 3a. Figure 3c,d shows digital images of the traditional Xuan paper before and after the continuous accelerated heat aging for 1200 days which is equivalent to 10,000 years of natural aging, exhibiting a significant difference in the appearance of the traditional Xuan paper before and after the continuous accelerated heat aging time equivalent to 10,000 years of natural aging. The traditional Xuan paper after the continuous accelerated heat aging time equivalent to 10,000 years of natural aging exhibits a yellow-brown color with a low whiteness of only 26.1%. In contrast, the whiteness of the fire-resistant “Xuan paper” is as high as 90.7% after the continuous accelerated heat aging equivalent to 10,000 years of natural aging, which is 3.48 times that of the traditional Xuan paper after the continuous accelerated heat aging equivalent to 10,000 years of natural aging.

Figure 3b shows the results of tensile strength versus aging time of the traditional Xuan paper before and after different accelerated heat aging times of up to 1200 days, which is equivalent of 10,000 years of natural aging. The tensile strength of the traditional Xuan paper is 17.1 N/15 mm before the accelerated heat aging, and 8.8 N/15 mm after the continuous accelerated heat aging for 60 days, which is equivalent to 500 years of natural aging, and the retention rate of tensile strength of the traditional Xuan paper is 51.5% for 500 years of natural aging. With the increase in aging time, the tensile strength of the traditional Xuan paper continues to decrease. When the continuous accelerated heat aging time is 720 days, which is equivalent to 6000 years of natural aging, the tensile strength of the traditional Xuan paper decreases to 1.2 N/15 mm with a retention rate of as low as 7%. As the continuous accelerated heat aging time increases to 1200 days, which is equivalent to 10,000 years of natural aging, the tensile strength of the traditional Xuan paper further decreases to 0.8 N/15 mm with a very low retention rate of only 4.7%, and the mechanical strength of the traditional Xuan paper has been almost completely lost, and the paper becomes very weak and brittle, and easily breaks when it is gently bending.

**Figure 4:**
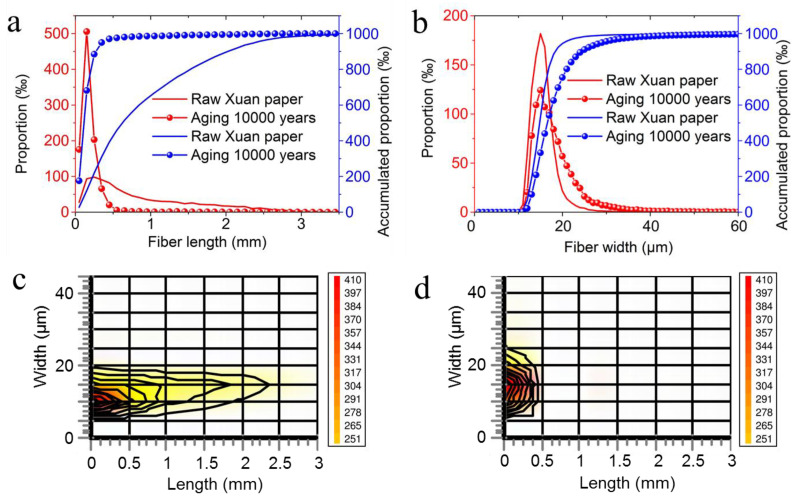
Characterization of cellulose fiber length distribution and fiber width distribution of the traditional Chinese Xuan paper before and after the continuous accelerated heat aging for 1200 days: (**a**) fiber length distribution curves; (**b**) fiber width distribution curves; and (**c**,**d**) width–length distributions of the traditional Chinese Xuan paper before (**c**) and after (**d**) the continuous accelerated heat aging for 1200 days.

Changes in the whiteness and tensile strength of the traditional Chinese Xuan paper are apparent phenomena: why do they occur during the continuous accelerated aging process? We investigated the composition, morphology, and length and width distributions of cellulose fibers before and after the continuous accelerated heat aging. As the aging time continues to extend, the fragmentation of cellulose fibers becomes more and more severe. The fragmentation and fracture of cellulose fibers lead to the decrease in fiber length and width, which are supported by the analytical results of cellulose fiber tests, as shown in Figure 4. Figure 4 shows a characterization of the cellulose fiber length distribution and fiber width distribution curves of the traditional Chinese Xuan paper before and after the continuous accelerated heat aging for 1200 days, equivalent to 10,000 years of natural aging. Before the accelerated heat aging, the fiber lengths of the traditional Xuan paper have a wide distribution range up to about 2.5 mm (Figure 4c). However, after the continuous accelerated heat aging time of 1200 days, equivalent to 10,000 years of natural aging, the fiber lengths of the traditional Xuan paper decrease significantly, exhibiting a length distribution range of up to about 0.5 mm (Figure 4d). The accelerated heat aging has a little influence in fiber width, ranging from 5 to 25 μm after the continuous accelerated heat aging for 1200 days, equivalent to 10,000 years of natural aging (Figure 4d).

From the above experimental results, it can be seen that the accelerated heat aging has a great influence in fiber length, leading to the significant shortening of cellulose fibers, which is a direct reason for the significantly decreased tensile strength of the traditional Chinese Xuan paper during the continuous accelerated heat aging process. These experimental results provide direct evidence for the significantly deteriorated properties and the related mechanism of the traditional Chinese Xuan paper during the continuous accelerated heat aging process. In contrast, the fire-resistant “Xuan paper” exhibits much better performance and superior properties compared with the traditional Chinese Xuan paper during the continuous accelerated heat aging process.

**Figure 5:**
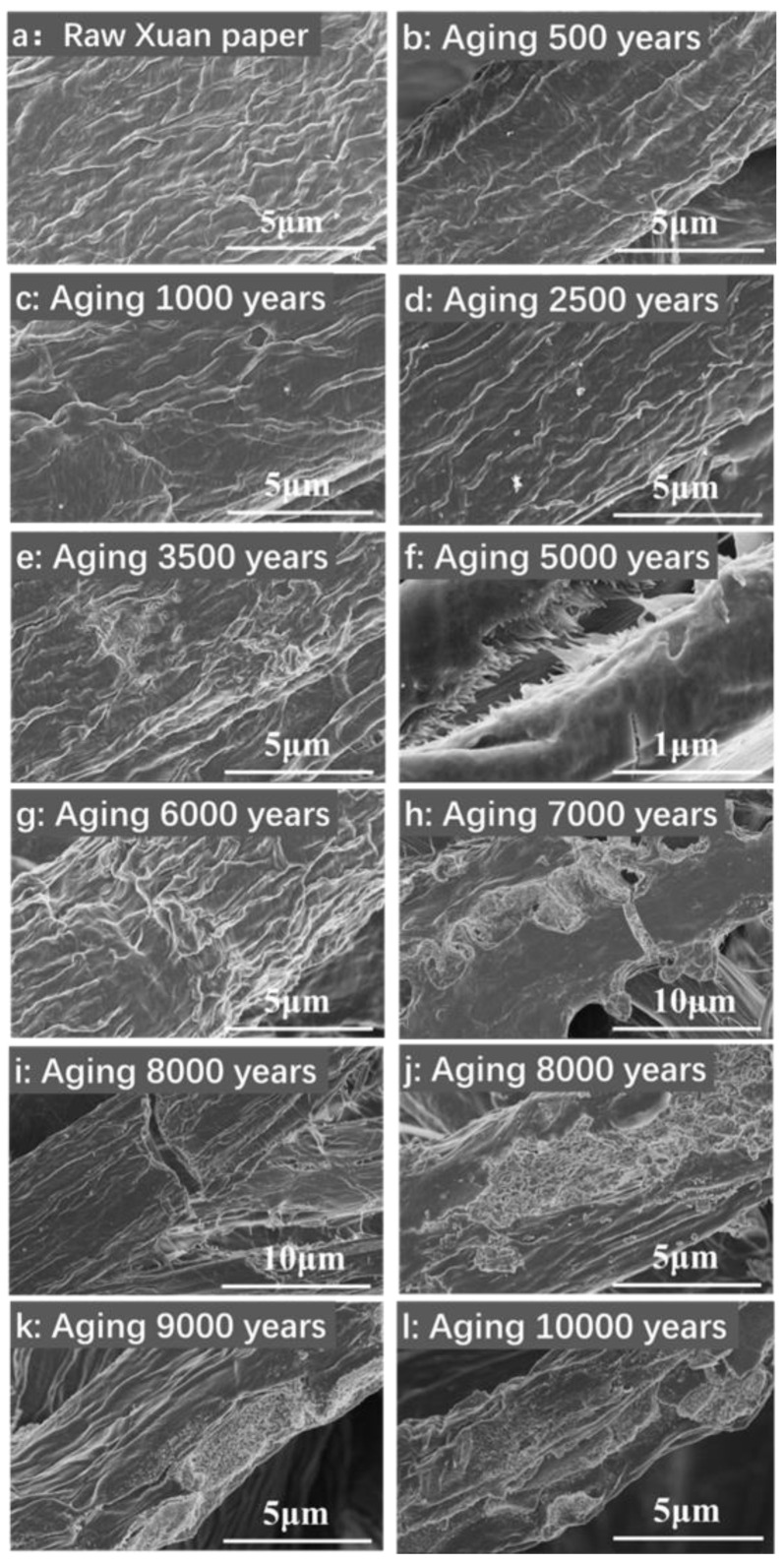
SEM images of the traditional Chinese Xuan paper before and after different accelerated heat aging times.

Figure 5a shows a SEM image of the traditional Chinese Xuan paper before the accelerated heat aging. When the continuous accelerated heat aging time is less than 2500 years of natural aging, significant changes in the morphology of cellulose fibers in the traditional Xuan paper are not observed (Figure 5b–d). However, when the continuous accelerated heat aging time is equivalent to 3500 years of natural aging, the obvious fragmentation phenomenon of cellulose fibers on the surface of the traditional Xuan paper appears (Figure 5e). When the continuous accelerated heat aging time is equivalent to 5000 years of natural aging, large-sized tearing gaps appear on the surface of the traditional Xuan paper (Figure 5f). When the continuous accelerated heat aging times are equivalent to 6000~8000 years of natural aging, severe damages such as cracks, holes, broken fibers, and fragmentation are observed on the surface of the traditional Xuan paper, as shown in Figure 5g–j.

**Figure 6:**
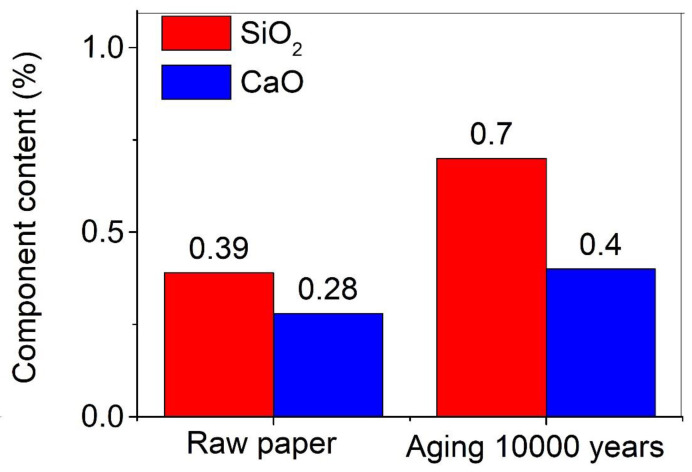
SiO_2_ and CaO contents of the traditional Xuan paper before and after the continuous accelerated heat aging for 1200 days, equivalent to 10,000 years of natural aging.

The traditional Xuan paper has good durability, which is attributed to its unique building materials and handmade manufacturing process, including more than 100 steps. The traditional Xuan paper is mainly made of the bark of *Pteroceltis tatarinowii*, a kind of cellulose fiber, which is an organic compound with the formula (C_6_H_10_O_5_)_n_, a kind of polysaccharide consisting of a linear chain of several hundred to thousands of β(1–4)-linked D-glucose units. *Pteroceltis tatarinowii* is produced in Jing County, Anhui Province, China. The local geological limestone leads to the deposition of a layer of limestone particles on the bark of *Pteroceltis tatarinowii*, which can neutralize acidic compounds produced by cellulose hydrolysis and acids from the environment. This is one of the important reasons for the good durability of the traditional Xuan paper. The components of SiO_2_ (0.39 wt.%) and CaO (0.28 wt.%) are detected, in addition to the main elements of C, H, and O in the traditional Chinese Xuan paper (Figure 6). However, after a long period of the accelerated heat aging for 1200 days, equivalent to 10,000 years of natural aging, the contents of SiO_2_ and CaO increase to 0.70 wt.% and 0.40 wt.%, respectively. Although the traditional Chinese Xuan paper is the most durable paper, the structure and properties of the traditional Chinese Xuan paper still deteriorate significantly with the extension of the aging time.

**Figure 7:**
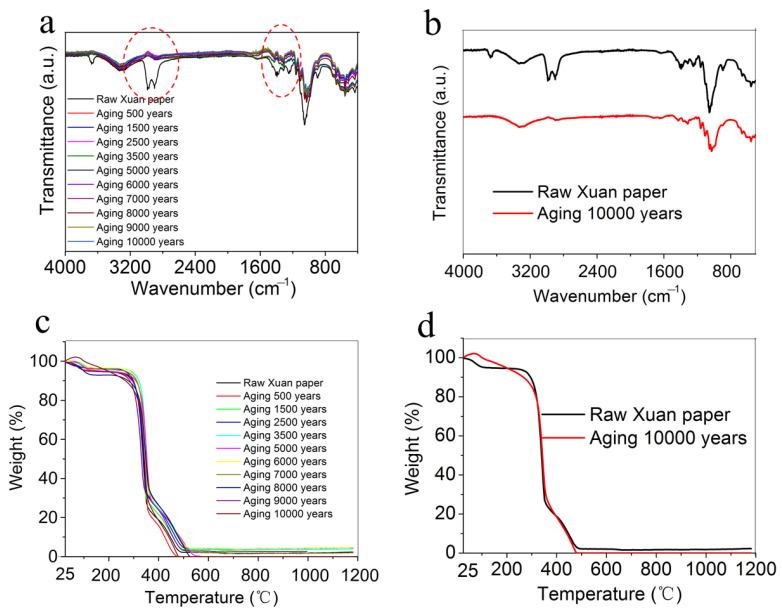
FTIR spectra (**a**,**b**) and TG curves (**c**,**d**) of the traditional Chinese Xuan paper before and after the continuous accelerated heat aging for different times.

The cellulose fibers are the main raw material of the traditional Chinese Xuan paper, which contain C–O–C, C–O, C–H, and O–H groups. The organic nature of cellulose fibers makes the traditional Chinese Xuan paper highly flammable. Figure 7a,b shows the Fourier-transform infrared (FTIR) spectra of the traditional Chinese Xuan paper before and after the continuous accelerated heat aging for different times of up to 1200 days, equivalent to 10,000 years of natural aging. The absorption peaks at 3337 cm^−1^, 1408 cm^−1^, 1338 cm^−1^, and 657 cm^−1^ are attributed to the stretching vibration absorption peak, in-plane vibration absorption peak, in-plane bending absorption peak, and out-of-plane deformation vibration absorption peak of the hydroxyl group (O–H), respectively. The absorption peaks at 2993 cm^−1^ and 2901 cm^−1^ are attributed to the stretching vibration absorption peaks of methylene. The peak located at 1670 cm^−1^ corresponds to C = C double bond stretching. The absorption peak at 1377 cm^−1^ is attributed to the bending vibration absorption peak of methylene. The absorption peaks at 1310 cm^−1^ is attributed to the shear vibration absorption peak of methylene. The absorption peaks at 1250 cm^−1^, 1066 cm^−1^, 1057 cm^−1^, and 896 cm^−1^ are ascribed to vibration absorption peaks of in-plane and out-of-plane stretching deformation absorption peaks of the C–O–C. It can be seen from the FTIR spectra that the absorption peaks of 2993 cm^−1^, 2901 cm^−1^, 1377 cm^−1^, and 1250 cm^−1^ are significantly reduced after the continuous accelerated heat aging (as shown in the red dashed circle in Figure 7a), indicating that the C–H and C–O groups are seriously broken during the continuous accelerated heat process.

Thermogravimetric (TG) analysis was performed to evaluate the thermal stability of the traditional Chinese Xuan paper before and after the continuous accelerated heat aging for different times of up to 1200 days, equivalent to 10,000 years of natural aging, and the results are shown in Figure 7c,d. The weight loss below 300 °C is mainly due to the loss of adsorbed water. The weight loss in the temperature range of 300~500 °C is attributed to the thermal degradation of cellulose fibers. For all samples, the weight loss is almost close to 100% at temperatures above 500 °C, indicating that cellulose fibers are almost completely decomposed at temperatures above 500 °C. In addition, the TG curves of all samples are similar, indicating that the continuous accelerated heat aging has no significant effect on the thermal stability of the traditional Chinese Xuan paper.

**Figure 8:**
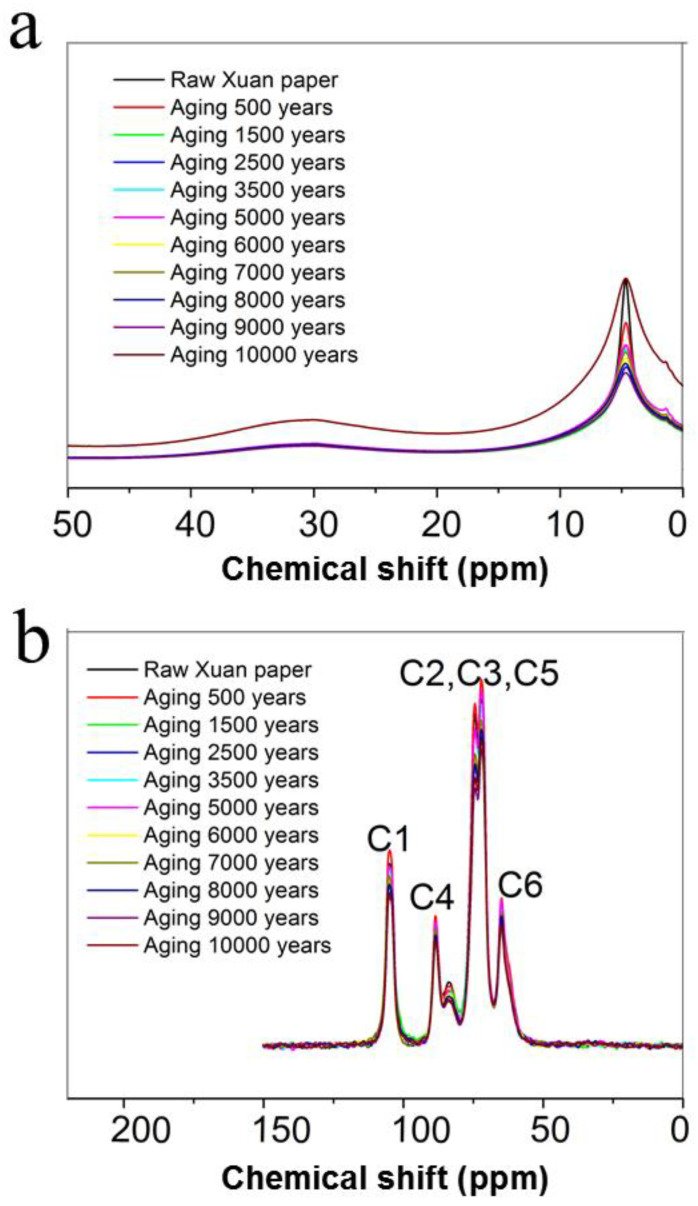
H–Nuclear magnetic resonance spectra (**a**) and C–nuclear magnetic resonance spectra (**b**) of the traditional Chinese Xuan paper before and after the continuous accelerated heat aging for different times.

Figure 8 shows the H-nuclear magnetic resonance (NMR) spectra and C–NMR spectra of the traditional Chinese Xuan paper before and after different accelerated heat aging times of up to 1200 days, equivalent to 10,000 years of natural aging. No obvious change is observed in spite of the severe degradation of cellulose fibers during the continuous accelerated heat aging process. The C NMR and H NMR spectra of the traditional Chinese Xuan paper reflect the environments of C and H atoms. In the H NMR spectra (Figure 8a), the chemical shift of a weak peak at 29.81 ppm corresponds to methylene groups. The proton peak at 4.67 ppm represents the hydroxyl proton at the C3 position of cellobiose, as well as proton peaks from the glucopyranose backbone and the amino proton. Figure 8b shows the C–NMR spectra of the traditional Chinese Xuan paper before and after different continuous accelerated heat aging times of up to 1200 days, equivalent to 10,000 years of natural aging. The chemical shifts of carbon at 60–70 ppm are attributed to the peak of C6. The chemical shifts of carbon at 70–81 ppm are attributed to the peaks of C2, C3, and C5, which are not bonded to glycosidic bonds, but on the glucose carbon ring. The chemical shifts of carbon at 81–93 ppm are attributed to the peak of C4. The C4 signal splits into two peaks, implying the signal peaks of cellulose C4 atoms in the amorphous and crystalline regions, located at 81–87 ppm and 87–93 ppm. The chemical shifts of carbon at 102–108 ppm are ascribed to the peak of C1 [35,36,37].

Therefore, although the traditional Chinese Xuan paper has already been renowned by its good durability among various cellulose-fiber-based paper materials, compared to the high stability of the fire-resistant Xuan paper, the traditional Chinese Xuan paper still shows an obvious deterioration in the structure and properties after the long-term aging. The cellulose fibers are the main component of the traditional Chinese Xuan paper, and their fiber length significantly decreases after the continuous long-term aging. Although no significant change can be observed in the morphology of cellulose fibers in the traditional Xuan paper during the accelerated heat aging for a relatively short period of time, the obvious fragmentation phenomenon of cellulose fibers appears after the accelerated heat aging time, equivalent to 3500 years of natural aging. Consistently, the FTIR results reveal the breakage of C–H and C–O bonds during the continuous accelerated heat aging. In the aging process, the cellulose, hemicellulose, and lignin within the traditional Chinese Xuan paper undergo oxidative degradation, resulting in the yellowing of the paper, breakage of C-C, C-H, and C-O bonds, and fracture of cellulose fibers. As a result, the traditional Chinese Xuan paper shows obvious decreased properties, for example, low retention rates of whiteness and tensile strength of 35.9% and 4.7%, respectively, after the continuous accelerated heat aging time of 1200 days, which is equivalent to 10,000 years of natural aging. The yellowing and brittleness of the traditional Chinese Xuan paper is unfavorable during the long-term preservation of books and important documents.

The distinct aging behaviors between the novel fire-resistant “Xuan paper” and traditional Chinese Xuan paper provide valuable insight into the design of highly durable paper materials. As for the traditional Chinese Xuan paper, although it enhances the durability through the use of the bark of *Pteroceltis tatarinowii*, which is deposited with limestone particles for neutralizing the acidic compounds from the hydrolysis of cellulose fibers, as well as by an elaborate handmade manufacturing process including more than 100 steps, it still cannot solve the problem of yellowing and mechanical performance degradation caused by the oxidative degradation and fragmentation phenomenon of cellulose fibers. This indicates that the aging behaviors of the traditional Chinese Xuan paper are primarily determined by cellulose fibers as its raw material, and this problem is difficult to change through reprocessing. In comparison, the novel fire-resistant “Xuan paper” is composed of all-inorganic components, which cannot be oxidized or degraded during its long-term storage in the ambient environment. As a result, the fire-resistant “Xuan paper” exhibits not only ultralong durability and superior anti-aging performance, but also high thermal stability and fire-resistant properties. Hence, this novel kind of fire-resistant “Xuan paper” is promising to be used in the protection of valuable art works and important paper documents for long-term preservation and protection from fire damage. Our work provides a pathway to designing highly durable “Xuan paper” by using inorganic fibers and inorganic adhesives.

## 3. Experimental Section

### 3.1. Materials and Chemicals

Chemicals such as CaCl_2_, NaH_2_PO_4_·2H_2_O, sodium oleate, and ethanol were purchased from Sinopharm Chemical Reagent Co., Ltd. (Shanghai, China), Glass fibers (GFs) were obtained commercially. The inorganic adhesive was prepared by the authors’ laboratory. The traditional unprocessed Xuan paper was used in the experiments and it was obtained from China Xuan paper Co., Ltd. (Xuancheng, China).

Ultralong HAP nanowires were synthesized by the calcium oleate precursor hydrothermal method previously reported by this research group [4]. The fire-resistant “Xuan paper” was prepared using ultralong HAP nanowires, glass fibers, and inorganic adhesive according to the method previously reported by this research group [4].

### 3.2. Characterization

The samples were characterized by scanning electron microscopy (SEM, Gemini 450, Zeiss, Oberkochen, Germany). The samples were coated with gold for 15 s before SEM observation. Fourier-transform infrared (FTIR) spectra of samples were recorded by the FTIR spectroscopy (FTIR–7600, Lambda Scientific, Edwardstown, Australia). The sample was ground with KBr and then pressed into the pellet for the transmittance FTIR test. Thermal properties of samples were obtained by thermogravimetric (TG) analysis (STA 409/PC, Netzsch, Selb, Germany) at the heating rate 10 °C min^−1^ in flowing air. The H–NMR and C–NMR spectra were recorded by the nuclear magnetic analyzer (Bruker 400M, Bruker, Fällanden, Switzerland). Fiber lengths and widths were measured by the fiber tester (L&W Fiber tester plus, L&W, Zurich, Switzerland). The tensile strengths of samples were tested by a universal testing machine (DRK 101A, Drick, Jinan, China) at room temperature. The contents of CaO and SiO_2_ in the traditional Xuan paper were determined by X-ray fluorescence spectroscopy (AXIOS, PANalytical, Amstelplein, The Netherlands). The tensile strength and whiteness tests of the paper samples were conducted according to the TAPPI standards.

### 3.3. Durability Evaluation

In this work, the continuous accelerated heat aging method (according to the standard of the Technical Association of the Pulp and Paper Industry of USA (TAPPI)) was used to evaluate the durability of paper samples. In this method, the paper sheets were put into an electric oven and maintained at a constant temperature of 105 ± 2 °C. Accelerated heating aging for 72 h corresponds to natural aging for 25 years in the ambient environment. After continuous accelerated heating aging for different times, the properties of the paper sheets were measured.

## 4. Conclusions

In this work, the traditional Chinese Xuan paper based on cellulose fibers and the fire-resistant “Xuan paper” based on ultralong HAP nanowires are evaluated by the continuous accelerated heat aging in the lab for 1200 days, which is equivalent to 10,000 years of natural aging in the ambient environment. The experimental results indicate that the whiteness and tensile strength of the traditional Chinese Xuan paper significantly decrease with the extension of the continuous accelerated heat aging time. The whiteness of the traditional Chinese Xuan paper is 72.8% before the continuous accelerated heat aging. When the continuous accelerated heat simulated aging time is 1200 days, which is equivalent to 10,000 years of natural aging, the whiteness of the traditional Xuan paper decreases to 26.1% with a retention rate of as low as 35.9%, indicating that the traditional Xuan paper exhibits a severe yellowing phenomenon during the continuous accelerated heat aging process. The tensile strength of the traditional Chinese Xuan paper is 17.1 N/15 mm before the continuous accelerated heat aging. As the continuous accelerated heat aging time increases to 1200 days, which is equivalent to 10,000 years of natural aging, the tensile strength of the traditional Xuan paper greatly decreases to 0.8 N/15 mm with a very low retention rate of only 4.7%, and the mechanical strength of the traditional Xuan paper has been almost completely lost, and the paper becomes very weak and brittle, and easily breaks when it is gently bending. With the extension of the continuous accelerated heat aging time, the fragmentation and fracture of cellulose fibers in the traditional Xuan paper lead to decreased fiber length, which is one direct reason for the significantly reduced tensile strength of the traditional Xuan paper. In contrast, no obvious change is observed for ultralong HAP nanowires and the properties of the fire-resistant “Xuan paper” after the continuous accelerated heat aging for 1200 days, which is equivalent to 10,000 years of natural aging. As a result, the fire-resistant “Xuan paper” exhibits outstanding super-durability, a superior preservation ability, and excellent properties such as whiteness and mechanical strength during the long-term aging process. After the continuous accelerated heat aging for 1200 days, which is equivalent to 10,000 years of natural aging, the whiteness of the fire-resistant “Xuan paper” slightly decreases from 92% to 90.7% with a retention rate of as high as 98.6%. Similarly, the tensile strength of the fire-resistant “Xuan paper” based on ultralong HAP nanowires is reduced from 8.3 to 7.9 N/15 mm after the accelerated heat aging, equivalent to 10,000 years of natural aging, with a high retention rate of 95.2%. The experimental results indicate that the fire-resistant “Xuan paper” based on ultralong HAP nanowires exhibits excellent performance and still maintains superior properties even after the accelerated heat aging equivalent to 10,000 years of natural aging in the ambient environment.

## Data Availability

The data presented in this study are available on reasonable request from the corresponding author.
